# Transcutaneous Spinal Cord Stimulation Enables Recovery of Walking in Children with Acute Flaccid Myelitis

**DOI:** 10.3390/children11091116

**Published:** 2024-09-12

**Authors:** Elizabeth Neighbors, Lia Brunn, Agostina Casamento-Moran, Rebecca Martin

**Affiliations:** 1 International Center for Spinal Cord Injury, Hugo W. Moser Research Institute at Kennedy Krieger Institute, Baltimore, MD 21205, USA; brunn@kennedykrieger.org (L.B.); martinre@kennedykrieger.org (R.M.); 2 Department of Biomedical Engineering; Johns Hopkins University School of Medicine, Baltimore, MD 21205, USA; 3 Department of Physical Medicine and Rehabilitation, Johns Hopkins University School of Medicine, Baltimore, MD 21205, USA

**Keywords:** acute flaccid myelitis, spinal cord injury, pediatrics, neuromodulation, transcutaneous spinal cord stimulation, gait, functional outcomes, rehabilitation, recovery

## Abstract

Background: Limited research exists for use of transcutaneous spinal stimulation (TSS) in pediatric spinal cord injuries (SCI) to improve walking outcomes, especially in children diagnosed with SCI secondary to acute flaccid myelitis (AFM). Objective: This case series demonstrates the feasibility and efficacy of TSS paired with gait training in children diagnosed with AFM. Methods: A total of 4 participants diagnosed with incomplete SCI secondary to AFM completed 22, 2-h therapy sessions over 5–8 weeks. TSS paired with body weight-supported treadmill training (BWSTT) was provided for the first 30 min of each session. Changes in walking function were assessed through the 6 min walk test (6MWT), Timed Up and Go (TUG), 10 m walk test (10MWT), and walking index for spinal cord injury II (WISCI-II). To assess safety and feasibility, pain, adverse events, and participant and therapist exertion were monitored. Results: All participants tolerated the TSS intervention without pain or an adverse response. Changes in the 6MWT exceeded the minimal clinically important difference (MCID) for three participants and WISCI-II exceeding the minimal detectable change (MDC) for two of the participants. Conclusions: These results demonstrate that TSS is a safe and clinically feasible intervention for pediatric patients with AFM and may supplement gait-based interventions to facilitate improvements in walking function.

## 1. Introduction

Acute flaccid myelitis (AFM) is found predominantly in children and is typically preceded by a viral infection [1,2,3,4,5]. It causes inflammation of the spinal cord, primarily affecting the ventral cord and anterior horn cells in the gray matter, resulting in rapid onset of paralysis [2,4,5]. Presentation is characterized by flaccid paralysis of one or more limbs and may include neck, trunk, and bulbar musculature with normal or near normal sensation [1,2,3,5]. Prognosis is variable, with up to 41% of children affected showing full recovery or minimal neurologic deficits after one year post onset, with recovery occurring distal to proximal [1,5]. However, many children have persistent deficits, including residual arm paralysis (75%), residual leg paralysis (60%), deficits in walking ability (58%), and persistent fatigue (52.7%) [1,6]. These impairments can significantly impact their independence with functional mobility, activities of daily living, and the ability to ambulate [4].

Acquired spinal cord injury (SCI), as in AFM, poses unique challenges to traditional rehabilitation [7,8,9]. Children require therapy to guide them through developmental patterns, often having to introduce skills not yet attained, while considering growth and maturation rather than restoring functions as in adult rehabilitation. Interventions that are well-studied in adults, such as body weight-supported treadmill training (BWSTT), have limited evidence in the pediatric population [10,11]. There are currently no guidelines specific to AFM regarding recovery-centered interventions, likely due to the small population, heterogeneity of presentation, and density of paralysis. A retrospective chart review published in 2020 reported significant functional improvement following inpatient treatment in this patient population [1]. This review by Hagen et al. showed that activity-based treatment interventions, including locomotor training, upper and lower extremity weight bearing, functional electrical stimulation, and developmentally appropriate mobility activities, did promote recovery and improved functional independence. However, this study was a retrospective review and did not target one specific intervention across all patients. Improvements in walking ability were not directly addressed, and while all participants showed strength improvements in a distal to proximal pattern, those improvements cannot be directly correlated to improved walking function. Additionally, most of their subjects were less than a year post diagnosis. There is a significant need for more information on effective treatments for children with chronic paralysis resulting from AFM.

Transcutaneous spinal cord stimulation (TSS) is a non-invasive therapeutic intervention that increases the excitability of spinal circuitry through activation of peripheral nerve roots, primarily dorsal root afferents [12,13,14,15]. The neuromodulatory input alters local motor thresholds to unmask volitional movement [12,13,14,15,16]. Research supports the use of TSS paired with treadmill training for improvements in postural control, gait mechanics, and motor output in adults with SCI [12,16,17,18]. In children with SCI, TSS has been found to improve trunk control, sitting posture, and hand function [19,20]. Additionally, studies addressing TSS in children with cerebral palsy (CP) indicate this intervention is feasible and shows promising efficacy for ambulation in pediatric care [21,22]. Given the spinal cord dysfunction associated with AFM paralysis, TSS could be a useful adjunct to traditional rehabilitation. However, to date, the combination of TSS and treadmill training has not been explored as an effective intervention to improve walking function within the AFM population. The current literature primarily focuses on the medical intervention and pharmaceutical treatment of AFM during acute diagnosis. Improvements in functional skills during inpatient care of AFM have been reported [1], but research evaluating therapeutic interventions and changes in function in chronic AFM is lacking. The purpose of this case series is to highlight the feasibility and efficacy of TSS paired with gait training in children diagnosed with AFM. Additionally, we aim to show improvements in walking function as demonstrated through increased walking speed, endurance, and independence. In doing so, we aim to elucidate trends in functional improvement within this population and present data to support effective treatment interventions for children with AFM.

## 2. Materials and Methods

### 2.1. Participants

Participants were recruited from the International Center for Spinal Cord Injury at Kennedy Krieger Institute. All participants were established outpatients and had previously participated in several rounds of activity-based rehabilitation. All four subjects included in this case series were participants in our ongoing research study with the following inclusion criteria: (1) 6–16 years of age; (2) >6 months post onset; (3) non-progressive spinal cord injury; (4) neurological injury level above T10; (5) tolerates upright position for >30 min; (6) able to advance one lower extremity in standing without physical assistance; (7) medically stable; and (8) able to comply with International Standards for Neurological Classification in Spinal Cord Injury (ISNCSCI). Exclusion criteria included the following: (1) active wounds; (2) range of motion (ROM) that limited gait training; (3) cardiac pacemaker/defibrillator; (4) active cancer diagnosis; (5) absent lower extremity reflexes; or (6) pregnancy. All participants were screened at baseline to ensure they met all inclusion criteria. The study was reviewed by the International Review Board (IRB) of Kennedy Krieger and Johns Hopkins Medical Institution and written informed consent from parents and assent from participants was obtained for all subjects. The participants reported here are part of a larger randomized control trial which is ongoing at the time of publication.

This larger study included four children with AFM between the ages of 8 and 12. This case series features these four participants to highlight the utility of TSS, specifically for children with AFM. Participants were on average 71 months post onset of AFM, with diagnosis established through imaging showing the presence of a spinal cord lesion primarily located in the gray matter. Participants 1 and 2 presented with C4 American Spinal Injury Association (ASIA) Impairment Scale (AIS) D tetraplegia per the ISNCSCI. They both used a power wheelchair for community mobility and ambulated within the home using a posterior walker. Participant 2 also required bilateral ankle foot orthoses (AFOs). Participant 3 presented with T4 AIS D paraplegia and was a community ambulator using bilateral lofstrand crutches and a right knee ankle foot orthoses (KAFO). Participant 4 presented with C4 AIS D tetraplegia and used a power wheelchair for home and community mobility but ambulated short distances without an assistive device and a cervical collar. See Table 1 for participant demographics and initial ISNCSCI scores.

### 2.2. Design

This prospective case series is a within-participant, repeated measure design. Given the small N of this case series, outcome measures were replicated at multiple time points and participants’ performance was compared only to their own. All participants received 22 intervention sessions with 2 additional pre- and post-assessment sessions. A subset of the larger trial data is reported here to highlight the unique challenges and progress in participants with AFM. All participants presented here received the experimental conditions described below. Measures of walking function were assessed at baseline and prior to every sixth session. Feasibility, pain, skin response, treatment time and adverse event data were collected at each session. All assessments were completed without stimulation.

### 2.3. Intervention

#### 2.3.1. Gait Training

Participants completed 2 h sessions, 3–5 times per week for 5–8 weeks, each participating in a total of 22 intervention sessions. This treatment duration was selected to match other gait interventions that have established efficacy [13,23,24]. Following our previous published protocol [13], TSS was applied continuously for the first 30 min of gait training followed by 90 min of intervention without stimulation to capitalize on the priming and neuromodulation effects of TSS [25,26,27]. Interventions included 60 min of BWSTT followed by 60 additional minutes of strengthening, task practice, and gait-based interventions. Body weight support (BWS) was selected individually for each child to maximize weight bearing while optimizing the appropriate gait kinematics at the trunk and lower extremities [24], ranging from 10 to 60% in these participants. Treadmill speed was adjusted throughout the session, with slower speeds utilized to maximize patient independence and faster speeds selected to match age-appropriate walking speeds. Speeds varied between 1.0 and 2.4 mph among our participants. Treatment guidelines were provided to trained physical therapists and occupational therapists while allowing for the use of clinical expertise and judgment to address participant specific deficits.

#### 2.3.2. Transcutaneous Spinal Cord Stimulation (TSS)

TSS was applied using the Vectra Neo (Chattanooga: Hixson, TN, USA) to provide a symmetrical biphasic rectangular waveform, at 1 millisecond pulse width and a frequency of 50 Hz [13]. This frequency was selected to target motor training while maximizing tolerance to the stimulation [13,26,27]. Considering comfort was particularly important in this population, with intact sensation to ensure participation in the protocol and to allow for tolerance to higher intensity levels. The Vectra uses a conventional waveform, with no carrier frequency. Intensity was set to tolerance at a submotor threshold, with stimulation ranging from 10 to 27 milliamps (mA) among participants. A 5 cm × 10 cm oval electrode was placed vertically and centered between T11 and T12 spinous processes as a cathode. Two additional electrodes were applied on the lower abdomen as anodes to direct the stimulation anteriorly through the spine [13,28].

### 2.4. Outcome Measures

#### 2.4.1. Safety and Feasibility

To assess the safety and feasibility of walking with TSS as an intervention for children with AFM, we monitored adverse events, self-reported effort, and pain. Monitoring for adverse response and session completion was continuous throughout the study. Completion was defined as 90 min of active therapy within a 2 h session. Sessions were scheduled for 2 h to accommodate rest breaks and transitions while allowing patients to complete 90 min of intervention. The Borg Rate of Perceived Exertion (RPE) was recorded from the participant and therapist at the end of each session. The RPE is a subjective, ordinal scale to measure self-perceived effort during physical activity. Effort was reported on a scale of 0–10 with 0 being equivalent to no exertion at all and 10 being maximal effort [29]. If an RPE was rated above 8, activities were modified for the next session. Pain scores were measured post intervention using the Numeric Rating Scale for Pain (NRS for Pain). The NRS for Pain is a 10-point scale, with 0 being a rating of no pain at all and 10 being the worst pain possible [30]. If a pain score was above five, an assessment was conducted with activity modifications and referrals to nursing or medicine as necessary.

#### 2.4.2. Walking Function

Walking function tests were administered pre- and post intervention, as well as prior to every sixth session. To determine the impact of TSS on functional ambulation, participants were assessed via the 6-Minute Walk Test (6MWT), 10-Meter Walk Test (10MWT), and the Timed Up and Go (TUG). These measures assessed multiple dimensions of gait, including speed and endurance, as suggested by the National Institute on Disability and Rehabilitation Research and the Common Data Elements recommended by the National Institute of Neurological Disease and Stroke and the National Institute of Health [31]. Additionally, the Walking Index for Spinal Cord Injury II (WISCI-II) was used to determine changes in required adaptive equipment and bracing during ambulation to demonstrate improved walking ability and independence. The WISCI-II is an impairment-related ordinal scale, where a higher number indicates more independence. These walking measures are widely used with good test–retest reliability, interobserver reliability, and construct validity in pediatric ambulatory SCI [31,32,33,34,35]. Though not validated for children under 13, the WISCI-II is recommended for use in this population by the National Institutes of Health and National Institute for Neurological Disease and Stroke Common Data Elements.

#### 2.4.3. Neurological Function and Quality of Life

The ISNCSCI was used to assess neurological change. The 36-item Short Form Survey (SF-36) was administered to gain insights into the participants’ overall health, participation, and quality of life. Both assessments were conducted at pre- and post-assessment sessions.

### 2.5. Data Analysis

As this was a small case series, changes are primarily reported at an individual level using only descriptive statistics and compared to the minimal clinically important difference (MCID) or the minimal detectable change (MDC) for each outcome measure. The MCID/MDCs used in this study are 45.8 m for the 6MWT [36], 0.06 m/s for the 10MWT [36,37], 10.8 s for the TUG [36], and a score change of 1 for the WISCI-II [38].

## 3. Results

### 3.1. Feasibility Data

No participant reported pain related to the stimulation, at the stimulation sites, during or after stimulation. There were some reports of pain related to the walking itself, at the joint or related to orthotic use. Participant 1 reported mild ankle discomfort during the initial three sessions. X-rays were completed as a precaution and were negative for fracture. The participant reported a 0/10 rating for the remainder of the study. Participant 2 reported 6/10 pain in one session due to orthotic discomfort, which resolved with modification of the orthotic. Participant 3 reported 0/10 pain in all sessions. Participant 4 reported 3/10 pain in lower back during a tall kneeling intervention, which resolved immediately following a change in position. There was a 98.48% session completion rate, showing good tolerance to the protocol. The average participant RPE was 5.87/10, with an average therapist RPE of 3.49/10. This therapist RPE translates to light to moderate activity. This indicates that incorporating TSS into gait-training interventions does increase the intensity for the participant, but does not increase the clinician’s burden, making it a feasible tool for clinical use.

No significant adverse events (e.g., skin burns, autonomic dysfunction, falls, or bowel and bladder issues) were identified or reported in these participants. Mild blanchable skin erythema did occur at the electrode site as is expected with surface electrical stimulation. This erythema was temporary and did not impact intervention.

### 3.2. Preliminary Effectiveness Data

#### 3.2.1. Walking Measures

**Figure 1:**
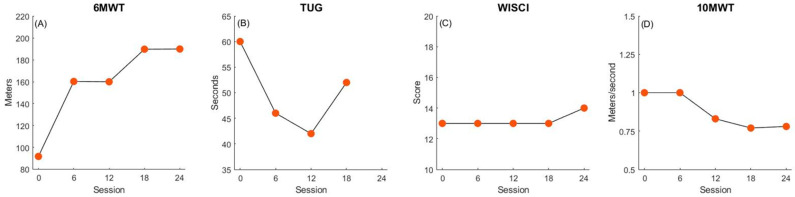
Participant 1 changes in walking function. Panels depict improvements in walking endurance (**A**), balance and mobility (**B**), walking quality (**C**) and walking speed (**D**) over the course of treatment.

Participant 1 demonstrated improved walking endurance measured by the 6MWT (+98.3 m, Figure 1A). He showed substantial improvements in TUG scores from enrollment through to the fourth assessment date (−8 s, Figure 1B). At the post-assessment visit, he was unable to stand from sitting, as required by the TUG. This was related to fatigue at this visit and did not remain a persistent problem. Improvements were also noted on the WISCI-II by 1 point, showing improved independence with functional ambulation (Figure 1C). His 6MWT and WISCI-II improvements exceeded the corresponding MCID/MDC (Figure 5). He did not show improvements in his 10MWT over the course of the study (−0.2 m/s).

**Figure 2:**
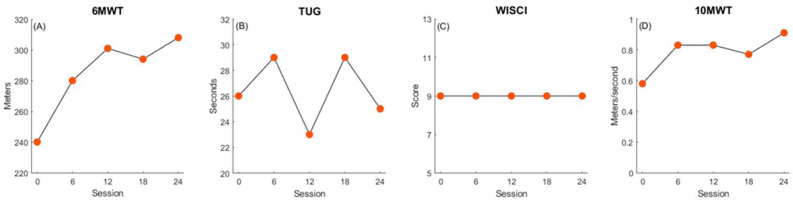
Participant 2 changes in walking function. Panels depict improvements in walking endurance (**A**), balance and mobility (**B**), walking quality (**C**), and walking speed (**D**) over the course of treatment.

Participant 2 increased walking endurance and speed at a greater magnitude than the MCID, demonstrated by improved scores on the 6MWT (+68 m, Figure 2A) and 10MWT (+19 m/s). TUG (+1 s, Figure 2B) and WISCI-II (+0, Figure 2C) scores showed minimal to no change pre- and post intervention.

**Figure 3:**
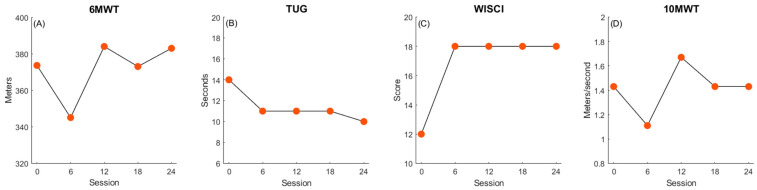
Participant 3 changes in walking function. Panels depict improvements in walking endurance (**A**), balance and mobility (**B**), walking quality (**C**), and walking speed (**D**) over the course of treatment.

Participant 3 improved scores in the 6MWT (+9.4 m, Figure 3A), TUG (−4 s, Figure 3B), and WISCI-II (+6, Figure 3C), exceeding the MDC for the WISCI-II and TUG (Figure 5). These positive changes translate to improvements in walking endurance, functional mobility, and assistance required during gait. She did not show improvements in the 10MWT (+0 m/s), possibly due to a ceiling effect as her walking speed was age appropriate when compared to children without physical disability prior to the intervention.

**Figure 4:**
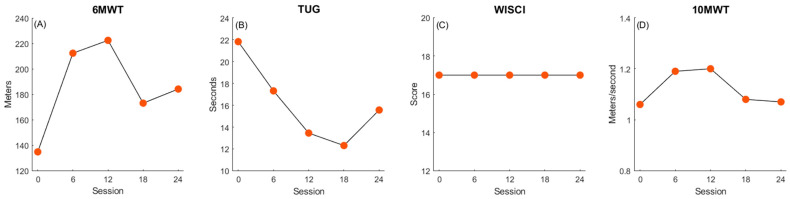
Participant 4 changes in walking function. Panels depict improvements in walking endurance (**A**), balance and mobility (**B**), walking quality (**C**) and walking speed, (**D**) over the course of treatment.

**Figure 5:**
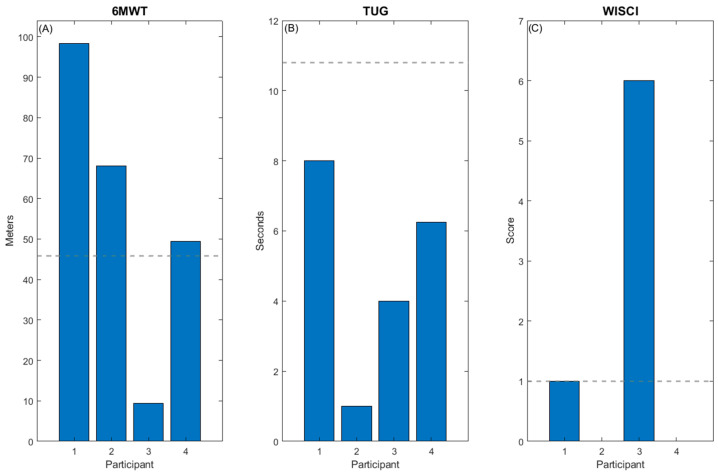
Change in walking function pre- and post intervention. Participants’ individual improvements in walking function pre- and post intervention are depicted in each panel (**A**) 6MWT, (**B**) TUG, (**C**) WISCI II. A dashed line indicates the MDC/MCID for each measure. Three out of the four participants demonstrate clinically significant change in the 6MWT, and two out of four in the WISCI II. No participant achieved the MDC for the TUG, indicating that the specificity of training likely had an effect.

Participant 4 showed significant improvement in his 6MWT (+49.4 m, Figure 4A) exceeding the MCID (Figure 5), as well as improved performance in the TUG (−6.25 s, Figure 4B). His 10MWT (+0.02 m/s) and his WISCI-II score (+0, Figure 4C) remained unchanged.

#### 3.2.2. Other Measures

Each participant reported improvements in self-perception of health and function as shown by scores on the SF-36. Specifically, participants increased their score in the role limitations due to the physical health category by an average of 43.75 points, with the individual results as follows: Participant 1 (+25 points), Participant 2 (+100 points), Participant 3 (+50 points), and Participant 4 (+0 points). Improvements in this category demonstrate decreased difficulty participating in age-appropriate activities and interaction within their environment. There were no significant changes in ISNCSCI scores for any participants pre- to post- intervention.

## 4. Discussion

This study demonstrates that TSS is safe and feasible when used in combination with BWSTT in children with non-traumatic spinal cord injury. Additionally, this initial evidence suggests that combinatorial TSS is efficacious in improving gait quality, speed, and endurance in pediatric onset AFM.

Walking endurance increased in all participants, with average change between pre- and post measures exceeding the MCID. Changes of greater magnitude were observed in participants with lower presenting status. It is encouraging that the combined intervention had an effect for participants who were not community ambulators. Three participants reported here demonstrated improvements exceeding the MCID for the 6MWT, suggesting greater endurance and activity tolerance. With goals of transitioning to community ambulation, improvement in walking endurance is a high-impact measure for these participants that translates to functional change. One participant’s walking endurance improved on a smaller scale. It is possible that as a community ambulator, there was a ceiling effect in the selected measures. This participant also saw stable 10MWT measures throughout the study; however, her walking speed was within normal limits when compared to age-appropriate normative data in healthy children [39]. Therefore, minimal change pre- and post intervention was expected.

The changes in selected outcomes does appear to reflect a specificity of training effect, in line with other gait-training paradigms. For example, participants’ change in the TUG was significantly impacted by their ability to stand from sitting. Sit-to-stand transfers were not addressed specifically, as training sessions focused primarily on walking-based interventions. Therefore, a greater magnitude of change was observed in walking function. Additionally, the TUG is not a standardized functional measure for pediatrics, as the chair height required for testing does not support a functional sit-to-stand in most children. This factor may also have contributed to the inconsistency in their TUG trials. These findings suggest the preferential improvement in spinally mediated patterned activity, rather than selective voluntary function when using TSS. The literature demonstrates the effects of epidural stimulation can evoke reciprocal step-like movements [18,25], and TSS is thought to increase the activation of spinal locomotor circuits to promote increased motor output and volitional movement [12,13,14,15,28]. This theory supports the use of TSS paired with gait-based interventions, and further supports the greater improvements noted in walking-based outcome measures.

Notably, we observed that all participants demonstrated improved trunk control, standing posture, and trunk and pelvic mechanics during gait following the intervention. These improvements likely contributed to the changes in gait speed and endurance found via the 10MWT and 6MWT. Though clinically useful, these outcomes are not sensitive enough to capture more subtle biomechanical changes. These more subtle changes, however, are particularly important for generalization of improvements and to prevent long-term dysfunction and consequences, like scoliosis. Observed changes in trunk function were consistent with reports from Keller et al. [19]

The SF-36 results showed improvement pre- and post intervention, showing an overall increase in participation and quality of life. However, consistent trends were noted—an increase in the role limitations due to physical health category, as well as a decrease in the energy/fatigue category. These results show that while our participants indicated a higher level of physical health, they also reported increased energy expenditure and fatigue post intervention. TSS increases spinal excitability, increasing motor recruitment and allowing patients to access volitional movement they may otherwise be unable to utilize [12,13,14,16]. This increased excitability facilitates increased muscle firing, and it would be reasonable to see an increase in fatigue immediately following intervention. With an increase in physical performance in function, it can be expected to see an increase in energy expenditure. While the SF-36 is not a common outcome measure within the pediatric population, we felt insight into our participant’s perception of their own health and physical functioning was important to evaluating the success of our intervention. The SF-36 is validated for use in adults with spinal cord injury and is easily accessible for clinicians for continued follow-up in an outpatient setting. All participants were able to verbalize understanding of the questions included in the SF-36 and answer to the best of their ability without parental influence.

Stimulation and session duration were selected based on previously established research demonstrating efficacy in the use of TSS to improve walking function in adults [12,13]. This protocol supports priming of the nervous system prior to overground training, while minimizing the risk of skin breakdown, maintaining patient engagement, and working within time constraints that are typical of an outpatient treatment session.

A common concern when using TSS in the pediatric population is children’s ability to tolerate stimulation, potentially limiting the feasibility of the intervention. This is of specific interest in the AFM population, as these patients typically present with intact sensation. All four participants highlighted in this case series had intact sensation, or hypersensitivity at their trunk and lower extremities as suggested during sensory testing through the ISNCSCI examination. The stimulation parameters were tolerated by all four participants, with no reported pain related to the stimulation. It is worth highlighting that the stimulation used here does not involve a carrier frequency. Eliminating this factor could make TSS more accessible to patients, as devices capable of carrier frequency are limited, without sacrificing tolerance and efficacy. Additionally, no adverse events or abnormal skin responses were identified during the intervention. These findings indicate that TSS is a safe and feasible intervention for pediatric patients with AFM, a non-traumatic SCI, even with intact sensation.

## 5. Limitations

As this is a case series, the sample size is very small. These participants were chosen to specifically highlight the AFM population; however, they are part of a larger randomized controlled trial. The ongoing study will have a larger and more representative sample of pediatric spinal cord injury with a comparative control group. While this case series does not include control group data, these participants were determined to be at their maximum functional capacity in traditional therapy, yet all showed improvements following the study protocol.

Most outcome measures in pediatrics look at broad function and participation. The options for accurately measuring impairment level changes are limited. While widely used clinically, the TUG, for example, is not validated for pediatric use. The requirement of a specific height chair does not consider the growth differences in pediatrics, potentially contributing to some of the test score variability. Self-report questionnaires can also be challenging to administer within the pediatric population, as the questions are often geared towards adult-specific activities such as work-related tasks and household chores. Questions can be challenging to understand and there is an increased risk of influential bias from parents or researchers when assisting a child with the survey. Additionally, the standard clinical measures used here were not sensitive enough to capture subtle biomechanical changes. Creating a robust outcomes dataset in pediatric research is challenging, and for this reason we selected the best available clinically relevant outcome measures.

## 6. Conclusions

Our findings indicate that TSS used in conjunction with gait training is clinically feasible and safe for pediatric patients with AFM. Additionally, we present data here to support TSS as an effective addition to gait training for pediatric patients with incomplete spinal cord injury secondary to AFM. Limited research exists regarding effective interventions for the AFM population, especially those with chronic impairments. Our initial findings support that TSS may supplement activity-based interventions, facilitating improvements in patients with chronic impairments. This ongoing study will compare the use of this combined approach with traditional therapy alone to improve walking function within the pediatric population.

## Figures and Tables

**Table 1:** Demographics. Orthotics and assistive devices listed used at baseline.

Subject	Gender	Age (Years)	Time since Injury (Months)	Neurological Level	ASIA Classification	Orthotics	Assistive Device
01	M	12	59	C4	D	none	posterior walker
02	F	8	61	C4	D	bilateral AFOs	posterior walker
03	F	8	94	T4	D	right KAFO	bilateral lofstrand crutches
04	M	10	72	C4	D	cervical collar	none

## Data Availability

Requests for access to de-identified dataset should be addressed to the corresponding author to respect patient privacy.
